# A Technique for Augmenting Bone Graft in the Tibial Tunnel During Anterior Cruciate Ligament Reconstruction Using Single-Tendon Hamstring Autograft

**DOI:** 10.1016/j.eats.2025.103760

**Published:** 2025-07-26

**Authors:** Fahad AlHulaibi, Ahmed Altwejri, Zaid Alzaid, Watban AlWatban, Yazen Alalwani

**Affiliations:** a Dammam Medical Complex, Dammam, Saudi Arabia; b King Fahd University Hospital, Al Khobar, Saudi Arabia; c Imam Abdulrahman Bin Faisal University, Dammam, Saudi Arabia

## Abstract

This article presents a technique to enhance graft-tunnel integration in anterior cruciate ligament reconstruction using a single-tendon hamstring autograft. The method involves injecting a calcium sulfate–calcium phosphate composite (Pro-Dense Injectable Regenerative Graft; Stryker) into the residual space between the distal graft and the tibial cortex. After preparation of a quadrupled semitendinosus graft with cortical suspension devices at both ends and a tibial fixation button, anatomically positioned femoral and tibial tunnels are created, and the graft is passed and fixed. The injectable graft material is delivered under controlled conditions to fill the tibial-tunnel void and support biological healing. Early application of this technique has shown ease of use, adequate tunnel filling, and no extravasation, with postoperative imaging confirming proper positioning. This approach addresses a critical biological limitation in anterior cruciate ligament reconstruction by enhancing tibial-tunnel integration. Although early results are promising, further studies are needed to evaluate long-term outcomes, including tunnel widening, graft incorporation, and functional performance.

Anterior cruciate ligament (ACL) injuries represent one of the most common ligamentous disruptions of the knee joint and frequently occur in athletic populations engaged in activities requiring sudden directional changes, deceleration, and pivoting movements. The incidence of ACL injuries continues to rise, with an estimated 250,000 cases occurring each year in the United States alone.^1^ Surgical reconstruction remains the gold-standard treatment for ACL-deficient knees in active individuals, with several techniques having been advanced over the past several decades to optimize functional outcomes and enhance return to preinjury activity levels.^1^

Hamstring tendon autografts have gained widespread acceptance as a preferred graft choice because of their favorable biomechanical properties, reduced donor-site morbidity, and favorable outcomes compared with alternative graft options.^2^ The traditional double-bundle technique using both the semitendinosus and gracilis tendons has been supplemented by the emergence of single-tendon approaches, which preserve gracilis function while achieving comparable stability when appropriately applied.^3^^,^^4^

Despite advancements in surgical techniques and fixation methods, challenges persist in achieving high-quality graft integration and long-term tunnel stability.^5^ An important concern in ACL reconstruction is the biological integration of the soft-tissue graft within the osseous tunnels, especially at the tibial interface, where biomechanical forces are significant and graft-tunnel motion may affect the healing process.^6^ The tendon-bone interface represents a complex biological junction at which nonoptimal integration may lead to tunnel widening, graft laxity, and further progression into reconstruction failure.^7^

Whereas bone graft augmentation has been reserved for revision ACL reconstructions to address the compromised bone stock and tunnel expansion,^8^ limited attention has been directed toward its application in primary cases. Recent evidence suggests that improving the biological environment within the tibial tunnel may accelerate graft incorporation and improve long-term stability, even in primary reconstructions.^9^ However, standardized techniques for bone graft augmentation in primary ACL reconstruction cases remain inadequately described in the literature.

The objective of this technical article is to describe an approach for augmenting bone graft within the tibial tunnel during primary ACL reconstruction using a single-tendon hamstring autograft. The proposed technique addresses the targeted interface between the graft and tibial cortex, especially in cases in which tunnel dimensions create residual spaces that may impact graft integration. By optimizing the biological milieu within the tibial tunnel through strategic placement of injectable calcium phosphate bone graft substitute, this approach aims to optimize graft incorporation, reduce tunnel widening, and potentially improve outcomes.

## Surgical Technique

### Preoperative Planning

Evaluation begins with a focused history and physical examination to determine whether the case is a primary ACL tear or a failed reconstruction. Radiographs (standing anteroposterior/lateral and alignment views) are obtained to assess tibial slope and alignment. Magnetic resonance imaging confirms the ACL tear and identifies additional intra-articular pathology.

### Patient Positioning and Preparation

The patient is positioned supine with a lateral support and distal post. A tourniquet is applied to the proximal thigh. Prophylactic antibiotics and tranexamic acid are administered 30 minutes before incision. Examination under anesthesia assesses instability. The limb is prepared with alcohol and povidone-iodine, followed by standard sterile draping.

### Graft Harvesting and Preparation

**Fig 1:**
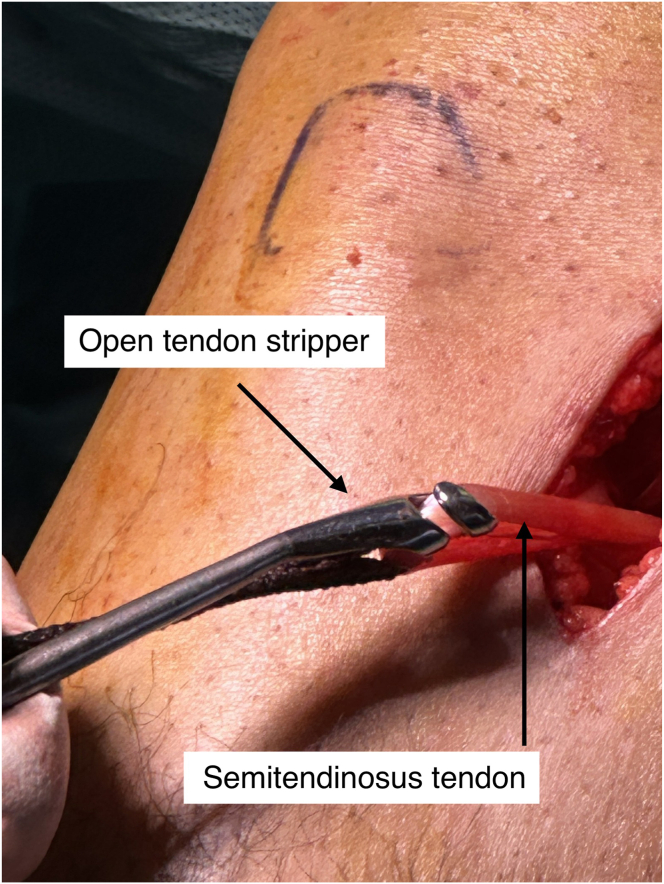
Harvesting of the semitendinosus tendon from the right knee with the patient in the supine position and the leg supported. A vertical incision is made over the pes anserinus, which is opened to retrieve the tendon using an open tendon stripper.

**Fig 2:**
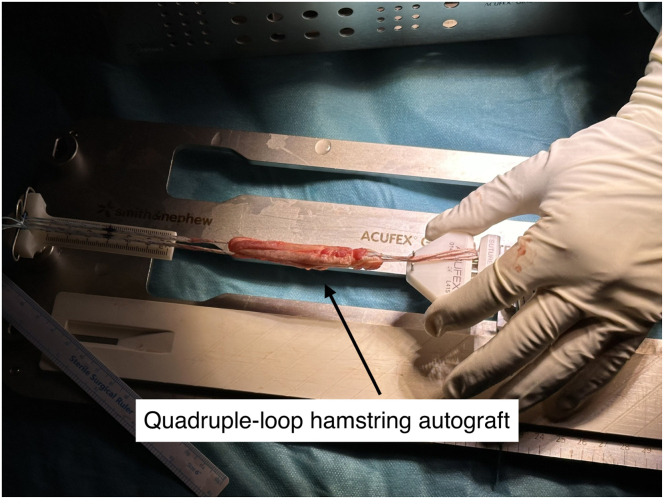
Back-table preparation of the quadruple-loop hamstring autograft. The graft is constructed with adjustable-loop cortical suspension devices (UltraButton) secured at both ends.

**Fig 3:**
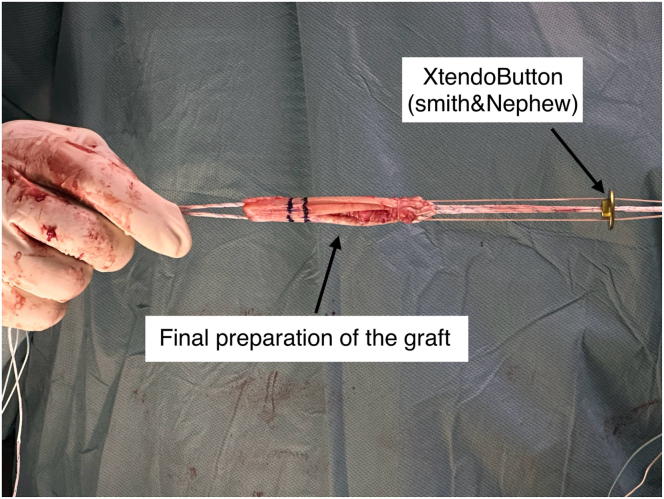
Final appearance of the prepared hamstring autograft, showing 20- and 25-mm markings from the femoral end to assist with accurate intra-socket positioning.

**Fig 4:**
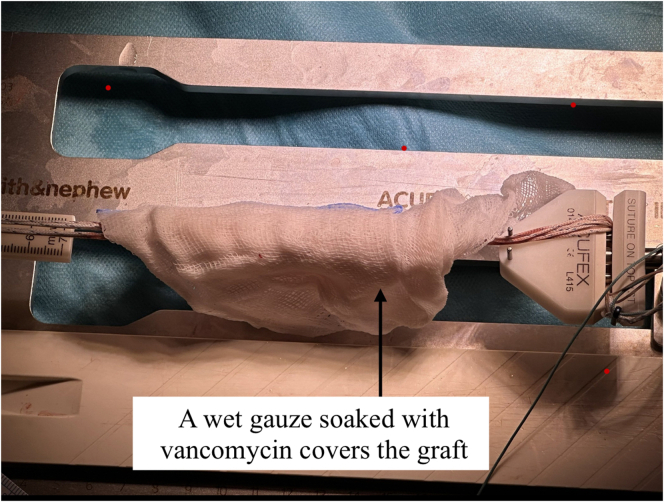
Tensioning and moistening of the hamstring autograft in vancomycin-soaked gauze prior to implantation.

A 3- to 4-cm vertical incision is made over the pes anserinus. The semitendinosus tendon is isolated, released, and harvested using an open tendon stripper (Fig 1). If the graft diameter is less than 7.5 mm, the gracilis tendon is also harvested. A quadrupled semitendinosus graft is constructed with adjustable-loop cortical suspension devices (UltraButton; Smith & Nephew) on both ends. A tibial-sided fixation button (XtendoButton; Smith & Nephew) is added (Figs 2 and 3). The graft is tensioned and stored in vancomycin-soaked gauze (Fig 4).

### Tunnel Preparation

After diagnostic arthroscopy and treatment of intra-articular pathology, the femoral tunnel is created first using the anteromedial-portal technique with the knee in greater than 120° flexion. A 2.7-mm guide pin is drilled through both cortices, followed by reaming to a 20- to 30-mm depth based on graft diameter (usually 8.5-9 mm).

**Fig 5:**
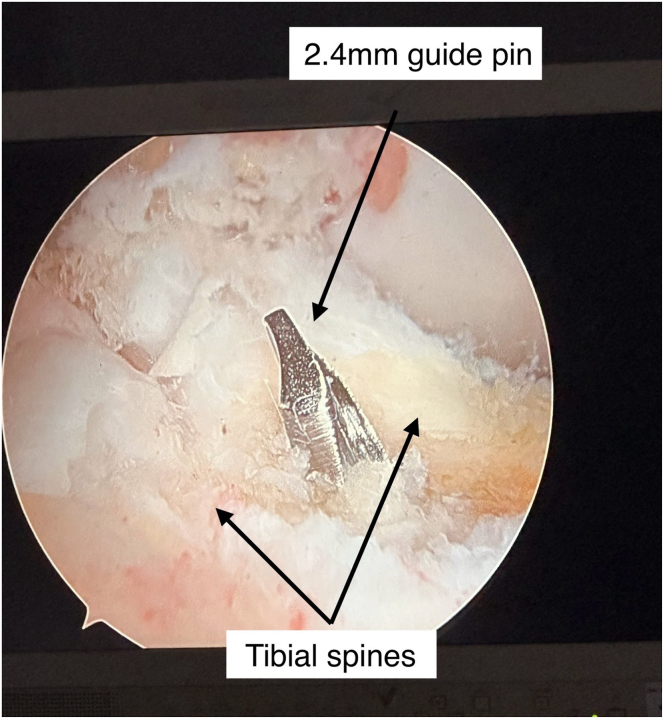
Arthroscopic view for the right knee from the anterolateral portal confirming 2.4-mm guide pin placement in the tibial tunnel at the native anterior cruciate ligament footprint.

**Fig 6:**
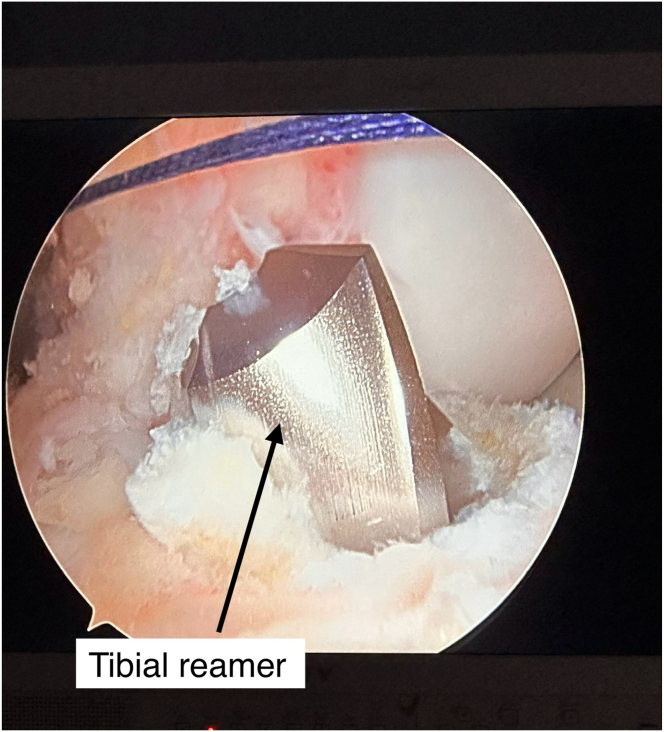
Tibial-tunnel reaming using a cannulated reamer based on graft diameter (typically 8.5-9 mm), under direct visualization, with the patient in the supine position.

The tibial tunnel is drilled at 57° relative to the plateau, targeting the native ACL footprint. The placement of a 2.4-mm guide pin is confirmed arthroscopically (Fig 5); the guide pin is then over-reamed using the appropriate reamer size according to graft diameter (Fig 6). Tunnel debridement is performed to smooth edges. The external tibial aperture is prepared and cleaned to fit the XtendoButton.

### Graft Passage, Fixation, and Tibial-Tunnel Bone Graft Augmentation

The graft is passed in a retrograde manner into the femoral tunnel using sutures. The femoral EndoButton (Smith & Nephew) is flipped over the lateral cortex as usual, and the graft is advanced to ensure 20-mm intra-socket occupancy. The graft is cycled and stabilized at 30° of flexion using a suspensory device and XtendoButton.

**Fig 7:**
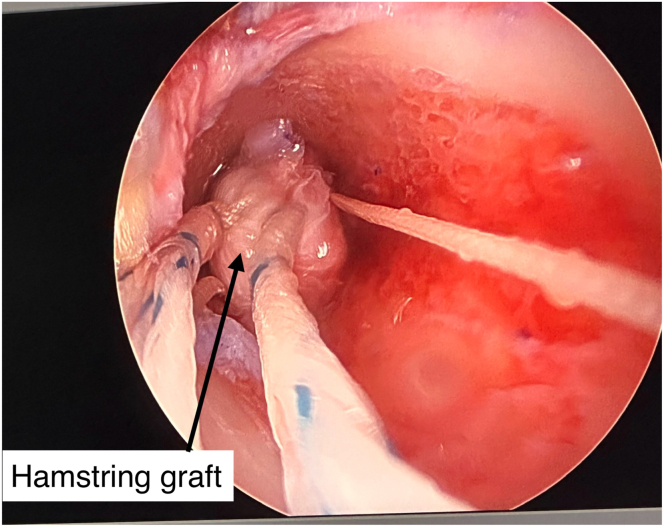
Arthroscopic view of the right tibial tunnel from the external cortex, showing the hamstring graft in place within the tunnel and the surrounding dead space to be filled with bone graft later.

**Fig 8:**
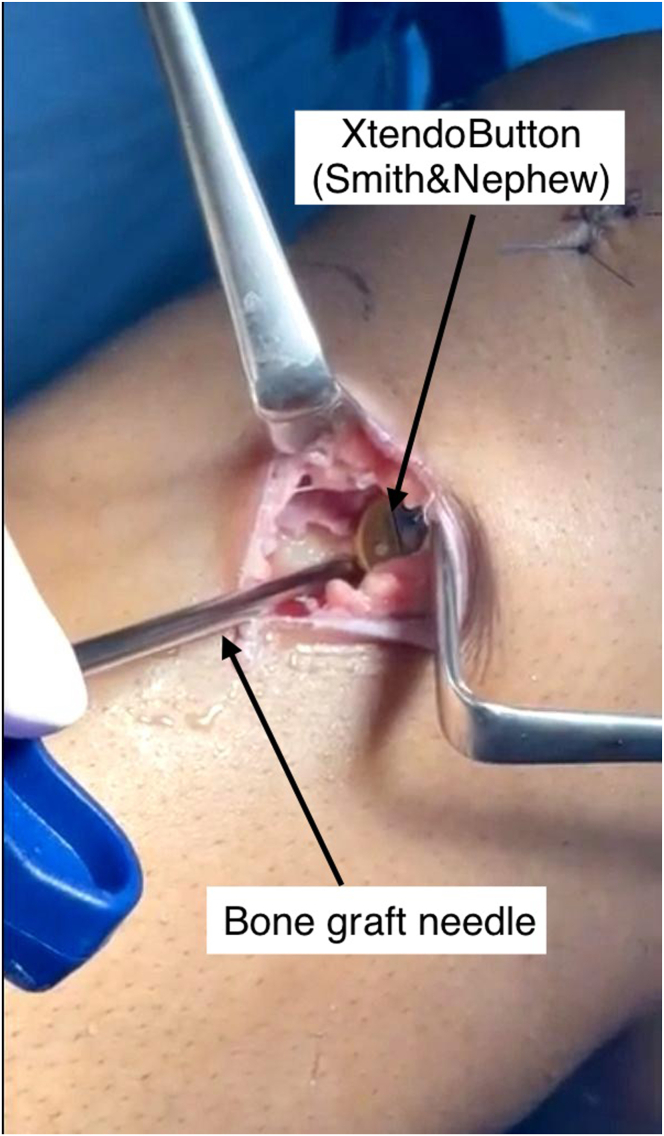
Intraoperative image showing bone graft injection into the tibial tunnel through the tibial incision in a right knee. The graft is used to fill the gap between the tendon and the cortical bone prior to final tensioning and fixation. The XtendoButton is visible in place before tensioning.

**Fig 9:**
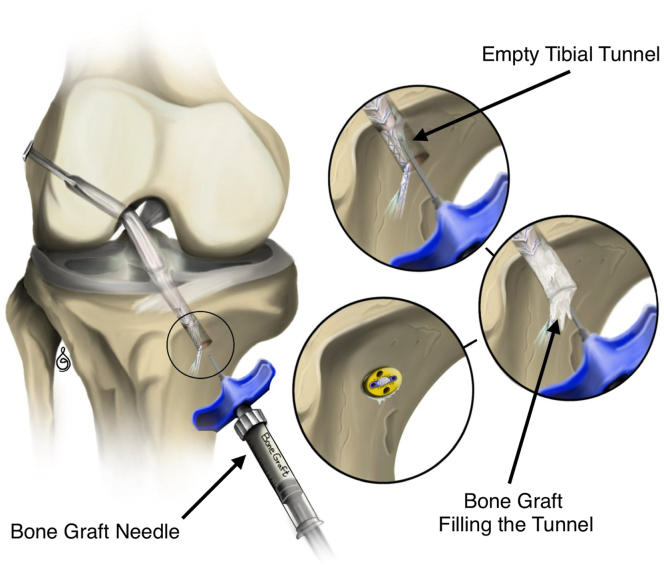
Injection of Pro-Dense (calcium sulfate–calcium phosphate) bone graft into the tibial tunnel to fill the void between the graft and cortical bone prior to final tensioning and fixation.

Before final tibial fixation (Fig 7), Pro-Dense (calcium sulfate–calcium phosphate; Stryker) is injected into the space between the distal graft and the tibial cortex to fill the void without extravasation (Figs 8 and 9). Excess graft material is removed, and the joint is irrigated before layered closure.

Postoperative radiographs confirm tunnel geometry, graft position, and bone graft placement. Video 1 illustrates all key steps of the technique.

## Discussion

Integration of soft-tissue grafts within osseous tunnels remains a key biological challenge in ACL reconstruction. This interface undergoes a healing process involving inflammation, cell proliferation, revascularization, and remodeling into a functional enthesis.^10^ The tunnels are particularly vulnerable to micromotion and shear forces, which can impair this process and lead to tunnel widening, graft laxity, and poorer outcomes.^11^

Whereas bone graft augmentation has traditionally been reserved for revision ACL cases with bone loss or malposition,^9^ applying this concept to primary reconstructions shifts focus toward optimizing the biological environment from the start. Our technique builds on this idea by injecting a calcium sulfate–calcium phosphate composite (Pro-Dense) into the tibial tunnel to support tendon autograft integration.

Biomechanically, this approach fills the distal graft-tunnel gap—an area prone to micromotion—with an osteoconductive scaffold that promotes cellular ingrowth and matrix deposition.^12^ Biologically, the graft’s resorption profile aligns with tissue incorporation timelines, offering both early structural support and long-term remodeling.^13^ The injectable format allows precise delivery without affecting graft tension or fixation.

Evidence supports this strategy: Biological augmentation has shown improved histologic and biomechanical properties in animal studies,^9^ as well as reduced tunnel widening with enhanced ossification in human ACL reconstructions.^14^ The Pro-Dense composite offers compressive strength exceeding that of cancellous bone and achieves meaningful mechanical integration within 3 months.^15^

Despite promising early use, several questions remain. The optimal volume and distribution of graft material in the tibial tunnel are still undefined and may influence vascularity or healing outcomes. Long-term effects on tunnel remodeling require imaging follow-up. Comparative studies against standard techniques are also needed to assess functional performance, return to sport, and revision rates.

Tibial-tunnel bone grafting offers several advantages that support its use in biologically focused ACL reconstruction. As summarized in Tables 1 and 2, the technique promotes graft incorporation, reduces dead space, and may help minimize tunnel widening. It is relatively easy to perform, requires no specialized instruments, and accommodates a variety of injectable bone grafts. However, it adds cost and may increase operative time if not well coordinated. Potential pitfalls include extravasation, difficulty with injection timing, and inconsistent material availability. Careful technique, team coordination, and planning are essential to maximize the benefits and avoid common technical challenges.

**Table 1:** Summary of Key Technical Pearls and Common Pitfalls Encountered During Tibial-Tunnel Bone Graft Augmentation in ACL Reconstruction

Pitfalls	Pearls (to Avoid Pitfalls)
The graft is too long, limiting space for the bone graft.	Always measure the graft length before implantation; keep the total length ≤70 mm and ≥20 mm in the femoral tunnel.
The bone graft extravasates into the soft tissue around the pes anserinus.	Inject the bone graft under direct visualization and perform adequate irrigation before closure.
The bone graft is difficult to inject after the tibial button is deployed.	Complete bone graft injection **before** final graft tensioning and tibial fixation.
Overfilling the tunnel with the bone graft increases graft-tensioning resistance.	Avoid over-packing the tunnel; fill just enough to eliminate dead space without impeding graft tension.
Bone graft preparation increases the operative time.	Delegate graft preparation to assistants during tunnel creation to minimize delays.
There is a lack of consistency in bone graft product availability.	Ensure availability of any injectable bone graft substitute compatible with the technique.

ACL, anterior cruciate ligament.

**Table 2:** Advantages and Disadvantages of Tibial-Tunnel Bone Grafting in Primary ACL Reconstruction

Advantages
Fills the dead space in the tibial tunnel
Enhances graft incorporation and bone remodeling
Simple technique requiring no special surgical skills
May reduce tunnel widening over time (long-term benefit)
Disadvantages
May increase operative time
Bone graft material sometimes not readily available
Adds additional material cost
Requires coordination for preparation and injection
Risk of extravasation into surrounding soft tissue

ACL, anterior cruciate ligament.

Future research should include computed tomography–based tunnel analysis and magnetic resonance imaging evaluation of graft maturation. Exploring this technique in femoral tunnels, although technically demanding, may further enhance biological healing strategies. Alternatives such as platelet-rich plasma, bone marrow aspirate, and growth factors could also be considered.

Although this technique focuses on tibial-tunnel augmentation in single-tendon ACL reconstruction, its principles may extend to other graft types and fixation methods. Optimizing the tunnel biology represents a broad and impactful strategy to improve ACL reconstruction outcomes.

## Disclosures

The authors declare the following financial interests/personal relationships which may be considered as potential competing interests: F.A. reports employment with Dammam Medical Complex. A.A. reports board membership and employment with Dammam Medical Complex. All other authors (Z.A., W.A., Y.A.) declare that they have no known competing financial interests or personal relationships that could have appeared to influence the work reported in this paper.

## Supplementary Data

Video 1 Comprehensive surgical presentation of tibial-tunnel bone graft augmentation in single-tendon hamstring anterior cruciate ligament (ACL) reconstruction. The key steps of bone graft preparation and injection using a calcium-based composite (Pro-Dense) are outlined.

## References

[1] Silvers-Granelli H.J. (2021). Why female athletes injure their ACLs more frequently? What can we do to mitigate their risk?. Int J Sports Phys Ther.

[2] Nukuto K., Hoshino Y., Kataoka K., Kuroda R. (2023). Current development in surgical techniques, graft selection and additional procedures for anterior cruciate ligament injury: A path towards anatomic restoration and improved clinical outcomes—A narrative review. Orthop Rev (Pavia).

[3] Kern M., Love D., Cotter E.J., Postma W. (2016). Quadruple-bundle semitendinosus-gracilis graft technique for all-inside anterior cruciate ligament reconstruction. Arthrosc Tech.

[4] Matteucci A., Högberg J., Piussi R. (2024). Comparison of knee flexor strength recovery between semitendinosus alone versus semitendinosus with gracilis autograft for ACL reconstruction: A systematic review and meta-analysis. Knee Surg Sports Traumatol Arthrosc.

[5] McDermott E., DeFoor M.T., Blaber O.K., Aman Z.S., DePhillipo N.N., Dekker T.J. (2023). Biomechanical comparison of anterior cruciate ligament reconstruction fixation methods and implications on clinical outcomes. Orthop Rev (Pavia).

[6] Yao S., Cao M.D., He X., Fu B.S.C., Yung P.S.H. (2021). Biological modulations to facilitate graft healing in anterior cruciate ligament reconstruction (ACLR), when and where to apply? A systematic review. Knee.

[7] Wang H., He K., Cheng C.-K. (2024). The structure, biology, and mechanical function of tendon/ligament–bone interfaces. Tissue Eng Part B Rev.

[8] Wang C., Hu Y., Zhang S. (2021). Application of stem cell therapy for ACL graft regeneration. Stem Cells Int.

[9] Rodríguez-Merchán E.C. (2021). Anterior cruciate ligament reconstruction: Is biological augmentation beneficial?. Int J Mol Sci.

[10] Gögele C., Hahn J., Schulze-Tanzil G. (2023). Anatomical tissue engineering of the anterior cruciate ligament entheses. Int J Mol Sci.

[11] Vignos M.F., Smith C.R., Roth J.D. (2020). Anterior cruciate ligament graft tunnel placement and graft angle are primary determinants of internal knee mechanics after reconstructive surgery. Am J Sports Med.

[12] Götschi T., Hodel S., Kühne N. (2023). Osteoconductive scaffold placed at the femoral tunnel aperture in hamstring tendon ACL reconstruction: A randomized controlled trial. Am J Sports Med.

[13] Georgeanu V.A., Gingu O., Antoniac I.V., Manolea H.O. (2023). Current options and future perspectives on bone graft and biomaterials substitutes for bone repair, from clinical needs to advanced biomaterials research. Int J Mol Sci.

[14] Mutsuzaki H., Sakane M., Nakajima H., Ochiai N. (2020). Calcium phosphate‑hybridized tendon graft to reduce bone‑tunnel enlargement after ACL reconstruction. Bone Joint Res.

[15] Mak C.Y., Lui T.H. (2022). Arthroscopic revision of attenuated anterior cruciate ligament graft with enlarged bone tunnels using injectable bone graft substitute. Arthrosc Tech.

